# Physicochemical Properties and Drivers of Liking and Disliking for Cooked Rice Containing Various Types of Processed Whole Wheat

**DOI:** 10.3390/foods10071470

**Published:** 2021-06-25

**Authors:** Da-Been Lee, Mi-Ran Kim, Jeong-Ae Heo, Yang-Soo Byeon, Sang-Sook Kim

**Affiliations:** 1 Research Group of Food Processing, Korea Food Research Institute, Wanju-gun 55365, Korea; Lee.Da-been@kfri.re.kr (D.-B.L.); ranni1027@kfri.re.kr (M.-R.K.); 50024@kfri.re.kr (Y.-S.B.); 2 Technical Assistance Center, Korea Food Research Institute, Wanju-gun 55365, Korea; Heo.Jeongae@kfri.re.kr; 3 Department of Biotechnology, College of Life Sciences and Biotechnology, Korea University, Seoul 02841, Korea

**Keywords:** cooked rice, processed whole wheat, physicochemical properties, consumer acceptance, drivers of liking and disliking

## Abstract

For utilization of whole wheat (WW) in cooked rice products, WW was processed by four different methods (steeping (S_WW), milling (M_WW), enzymatic treatment (E_WW), and passing through a roll mill (1 mm) (R_WW)). Additionally, the physicochemical properties of cooked rice containing various processed wheat were investigated. The hardness of the cooked rice decreased significantly with R_WW and E_WW compared to WW. As a result of a consumer acceptance test, the cooked rice samples containing M_WW and E_WW with high liking scores frequently included ‘chewiness’ as a reason for liking, and the cooked rice with WW and S_WW was mentioned as being ‘too hard’ as a reason for disliking. The cooked rice with R_WW, which had the lowest liking score, was mentioned as having appearance characteristics such as ‘husk’, ‘clumpy appearance’, and ‘messy appearance’ as reasons for disliking. The overall results of this study suggest the inclusion of M_WW or E_WW with cooked rice considering health-related benefits and consumer acceptability.

## 1. Introduction

Rice is a major food grain in Korea, and 95% of it is a commercial staple food consumed in the form of cooked rice [1]. Various cooked rice products have been developed by mixing other grains. In particular, wheat, as the second major grain, is consumed at levels of approximately 32 kg per capita per year in Korea [2]. However, most wheat is imported from the United States, Australia, and Canada, and only approximately 2% of the total amount consumed is produced in Korea [2]. Wheat is rich in starch, protein, dietary fiber, minerals, phenolic compounds, and phytochemicals [3]. As interest in health increases, the demand and interest in whole wheat is increasing. The consumption of these processed products is effective in preventing adult diseases such as hypertension and diabetes. In particular, whole wheat contains fiber, vitamin B, vitamin E, iron, and magnesium, is particularly rich in food fiber and is known to lower the risk of obesity, stroke, heart disease, diabetes and colon cancer [4,5,6]. Additionally, whole wheat has a low glycemic index (GI), which is good for diabetes management and lowers blood cholesterol, reducing the risk of arteriosclerosis and hypercholesterolemia [7]. The benefits of eating whole grain foods are well known, but their use is limited because of the resulting low sensory quality for processed foods [8].

Food development is a consumer-oriented task, and understanding consumers’ preferences has become a key factor in the success of such research [9,10]. Therefore, it is important to understand how consumers perceive products [11]. Thus, obtaining consumer feedback on the sensory description of a product as an alternative to conventional sensory profiling has become of great interest over the past two decades [12]. The conventional approach used to understand consumers’ preferences is internal and external preference mapping, which combines descriptive data provided by trained panels with acceptance tests performed by consumers [13]. The preference mapping technique requires consumer testing by consumers for acceptability and quantitative descriptive analysis of sensory properties by trained panels, which is expensive and time-consuming [9,12]. Furthermore, the terms generated by the trained panel may differ from those used by consumers and can be difficult for consumers to understand [10,14]. Therefore, ten Kleij et al. [14] proposed a textual analysis of open-ended questions to complement the preference mapping technique. In many studies, free-comment responses explicitly written by consumers in the form of open-ended questions have been mainly used to reduce respondents’ complaints by allowing them to explain their responses to other questionnaire items [15]. Moreover, free-comment responses have a definite advantage of being easy to understand because consumers’ opinions are expressed in their own language and do not require deep thinking. In addition, it is possible to obtain rich information, including what the researcher did not predict [16].

The objectives of this study were to evaluate the consumer acceptability of processed whole wheat products for cooked rice with improved texture using various processing methods to find out the potential of entering the market. Additionally, functional activity and physicochemical properties were investigated. In addition to consumer acceptances, consumer perceptions were studied by applying two open-ended questions to identify the drivers of liking and disliking for cooked rice samples of cooked rice samples with various types of processed whole wheat.

## 2. Materials and Methods

### 2.1. Physicochemical Property

#### 2.1.1. Materials

The five types of cooked rice were prepared with 60% rice (Sindongjin, Yeonggwang, Jeonlanamdo, Korea, 2019) and 40% whole wheat or various types of processed whole wheat (Beakchal, Yeonggwang, Jeonlanamdo, Korea, 2018). Whole wheat (WW) was processed in four ways: steeping (S_WW), milling (M_WW), enzymatic treatment (E_WW), and passing through a roll mill (1 mm) (R_MM). The S_WW was WW steeped with tap water at 4 °C for 24 h; M_WW was WW milled to remove 5% bran using a pearling machine (2RSB-10FS, Kett, Tokyo, Japan); E_WW was WW treated with 5% viscozyme (Novozyme, Bagværd, Denmark) at 50 °C for 24 h; R_WW was WW passed three times through a roll mill with gaps of 2.0, 1.5 and 1.0 mm, sequentially, after steeping in tap water at 4 °C for 16 h.

#### 2.1.2. Cooked Rice with Whole Wheat

Rice (540 g) and wheat (360 g) were washed with water using a rice cleaner (PR7J, Aiho, Tokyo, Japan). Filltered water was added to the rice and wheat at a weight ratio of 1.6:1 (14% moisture basis) and, then, the mixture was cooked using an electric rice cooker (CRP-LHTR1010FWM, Cuckoo, Yangsan-si, Gyeongsangnam-do, Korea). Cooked rice in a bowl was stirred smoothly five times with a spoon and then cooled for 5 min at room temperature. The stirring and cooling procedures of the cooked rice were repeated twice.

#### 2.1.3. Physical Property Analysis of Cooked Rice with Whole Wheat

The moisture content of cooked rice samples containing processed WW was measured according to American Association of Cereal Chemists (AACC) Method 44-15A (AACC, 2010) using a dry oven (HK-DO1000F, Hankuk S & I Co., Hwaseong, Korea). Texture profile analysis (TPA) of cooked rice was performed based on the modified AACC Method 74-09 (AACC, 2010) with a Texture Analyzer TA-XT plus system (Stable Micro System Ltd., Haslemere, UK). The cooked rice (12 g) was placed into a cylindrical container (4 cm diameter × 1 cm length) and compressed by approximately 40% using a 20 mm plunger. The return distance was 30 mm, the return speed was 1.7 mm/s, and the contact force was 50 g. Hardness, adhesiveness, springiness, cohesiveness, chewiness, and resilience were determined from the two-cycle curves using Texture Export for Window (Stable Micro Systems, Godalming, UK). All physicochemical analyses were repeated three times.

#### 2.1.4. Starch Hydrolysis Index

The in vitro starch hydrolysis index (HI) of cooked rice samples containing processed WW was determined according to a modified method [17]. Two grams of freeze-dried cooked rice powder was mixed with 100 mL of 0.05 M sodium potassium phosphate buffer (pH 6.9). Then, 110 U pancreatic amylase (Type I-A, Sigma Aldrich, St. Louis, MO, USA), was added, and the mixture was incubated in a shaking incubator (120 rpm) at 37 °C. Next, 2 mL aliquots were added every 0, 30, 60, 90, 120 and 180 min, boiled at 95 °C for 5 min, and cooled on ice. The aliquots were centrifuged at 4000 rpm for 10 min, and the reducing sugar content in the supernatant was measured by a modified method from Somogyi [18]. For each starch hydrolysis curve, the area under the curve (AUC) was calculated using SigmaPlot 13.0 (Systat Software Inc., Point Richmond, CA, USA). Hydrolysis indexes were repeated three times and calculated with the following equation.

HI = (AUC test food/average AUC reference sample) × 100(1)

### 2.2. Consumer Acceptance Test

#### 2.2.1. Sample Preparation and Presentation

The cooked rice samples used in the consumer acceptance test were prepared in the same way as those used to assess the physicochemical properties (Section 2.1.2). The cooked rice samples (50 g) were placed in a bowl (85 × 50 mm, diameter × depth) with a lid using a stainless-steel scoop. The cooked rice samples were coded with three-digit random numbers and served at 50 ± 5 °C. The samples were stored in a heating cabinet (HA-DB3000, Hains Co., Incheon, Korea) to maintain the temperature throughout the evaluation session. The serving order of the samples was determined by a William Latin square design [19], and samples were presented in sequential monadic order to avoid carryover effects [20]. Filtered water at ambient temperature was provided to the consumers to cleanse the palate between samples.

#### 2.2.2. Cooked Rice with Whole Wheat

A total of 103 consumers were students and research scientists at the Korea Food Research Institute (KFRI) recruited through e-mails. The participants were 36% male and 64% female, with an average age of 34.3 years. Written consent was given by all consumers. The testing was carried out in the sensory laboratory of the Korea Food Research Institute (KFRI) equipped with individual booths. Ten to twelve consumers assessed in the experiment at one time and all consumers completed the experiment on the same day. In the consumer acceptance test, the consumers rated the overall appearance, odor, taste/flavor, and texture liking of the samples on a standard 9-point hedonic scale (1 = dislike extremely, 5 = neither like nor dislike, 9 = like extremely) [21]. After evaluating their levels of liking, the consumers were asked to answer 2 open questions, freely describing the reasons for liking and disliking each sample. The procedure followed the method explained by Symoneaux et al. [10]. Answering was not mandatory. In this way, the participants could express only reasons for liking, only reasons for disliking, both or none for each sample. Data collection was carried out with Compusense 5.0 software (Compusense Inc., Guelph, ON, Canada).

### 2.3. Statistical Analysis

#### 2.3.1. Physicochemical Properties

The data were analyzed using SPSS statistical software (version 21; SPSS Corp., Chicago, IL, USA). ANOVA followed by Duncan’s multiple range test was applied to determine significant differences.

#### 2.3.2. Consumer Acceptance Test

For the liking data, analysis of variance (ANOVA) using a general linear model (GLM) was performed to determine the effects of the sample as a fixed source of variation and panel as a random effect. When the effects were significant, significant differences were calculated using Duncan’s multiple range test (*p* < 0.05). Pearson correlation coefficients between liking attributes were also calculated.

The open-ended questions were qualitatively analyzed. The reasons for liking and disliking the samples described by the panelist were written in each column and were divided by semicolons (;). The frequency of descriptors was calculated by textual analysis. Next, terms with similar meanings were combined with representative words. For example, ‘hardness’, ‘firmness’, and ‘rigidity’ were merged to ‘hardness’. The synonyms were reviewed by three other native researchers for validation. After refining the descriptors for statistical analysis, attributes that were mentioned by more than 5% of the consumers for at least one sample were used for further analysis. Finally, a contingency table was generated with rows of samples and columns as reasons for liking and disliking. As recommended by Symoneaux et al. [10], chi-square analysis per cell was applied to the contingency table to identify statistically significant components within a matrix. Additionally, correspondence analysis (CA) was performed to visually summarize the relationship between samples and descriptions. According to the suggestion of Lê et al. [22], only the terms with cos2 values above 0.8 were shown in the perceptual map to distinguish the attributes that are highly correlated with dimensions 1 and 2.

The data were analyzed using SPSS statistical software (version 21; SPSS Corp., Chicago, IL, USA) and FactoMineR 2.3 [23] of R Studio 1.4.1103 [24] based on R statistical system 4.0.3 [25].

## 3. Results

### 3.1. Characteristics of Cooked Rice with Various Types of Processed Whole Wheat

The water content and TPA results of the cooked rice with various types of processed WW are shown in Table 1. The water content was the highest in the cooked rice with R_WW (62.27%), while it was the lowest in the cooked rice with WW (57.77%). The hardness of the cooked rice decreased significantly with R_WW and E_WW. The cohesiveness of cooked rice with E_WW and R_WW markedly increased. There was no significant difference in the HI values among cooked rice samples with various types of processed WW.

### 3.2. Consumer Acceptance Test

#### 3.2.1. Consumers’ Liking Scores

The ANOVA results showed that the cooked rice samples were different in terms of all liking attributes (*p* < 0.05). The mean scores of liking for cooked rice samples are presented in Table 2. Based on the consumer ratings, the overall liking for the cooked rice with M_WW (5.8) was the highest, while that with the cooked rice with R_WW (4.4) was the lowest. In particular, the appearance liking score of the cooked rice with R_WW (4.0), in which the bran of the wheat kernel surface was destroyed, was significantly lower than that of the other samples. The results of Pearson’s correlation analysis showed a high correlation between the overall liking and taste/flavor liking score (r = 0.76) and texture liking score (r = 0.74). The texture liking score clearly indicated the enhanced textural quality of the cooked rice with M_WW and E_WW.

#### 3.2.2. Drivers of Liking and Disliking

Consumers described 117 and 139 reasons for liking and disliking the samples, respectively. Three native researchers merged similar terms to representative words, resulting in 67 and 80 reasons for liking and disliking, respectively. Among these terms, 16 reasons for each like and dislike were mentioned at least 5% in one sample. The reasons for liking the samples were mainly terms such as ‘corn odor’, ‘taste’, ‘nutty flavor’, ‘odor’, and ‘chewiness’, which were described 563 times in total. The reasons for disliking the samples were frequently mentioned, including attributes such as ‘too watery’, ‘strong after-effect’, ‘too hard’, ‘roughness’, and ‘appearance’, a total of 642 times. In addition, 100 consumers answered no reason for liking, and 82 consumers answered no reason for disliking, regardless of the sample.

The chi-square per cell enables the identification of the more or less used attributes for samples [10]. From Table 3, it can be observed that the cooked rice with S_WW was more often considered ‘not watery’ than the other samples, but was not described as exhibiting ‘softness’. The cooked rice with E_WW received more mentions of ‘chewiness’ as a reason for liking than the other samples. In contrast, the cooked rice with R_WW had the lowest citation frequency for ‘chewiness’. As a result of describing the reasons for disliking samples, the cooked rice with R_WW was cited more often as having negative attributes related to appearance, such as ‘husk’, ‘clumpy appearance’, and ‘messy appearance’. The cooked rice with WW and S_WW had similar tendencies. In both samples, ‘too watery’ was mentioned less than expected, and ‘too hard’ was mentioned more than expected. In addition, the cooked rice with WW was mentioned as ‘feels undercooked’, and the cooked rice with S_WW had more ‘roughness’ mentions than other samples. Twenty-eight subjects even responded that there was ‘no liking reason’ for the cooked rice with WW. The cooked rice with M_WW was characterized by a higher citation frequency for ‘too watery’ and ‘too sticky’ and fewer citations for ‘roughness’ as dislikes. However, the twenty-four consumers responded that there was ‘no disliking reason’ for cooked rice with M_WW. The cooked rice containing E_WW was significantly less frequently referred to as ‘too hard’ for reasons of disliking.

**Figure 1:**
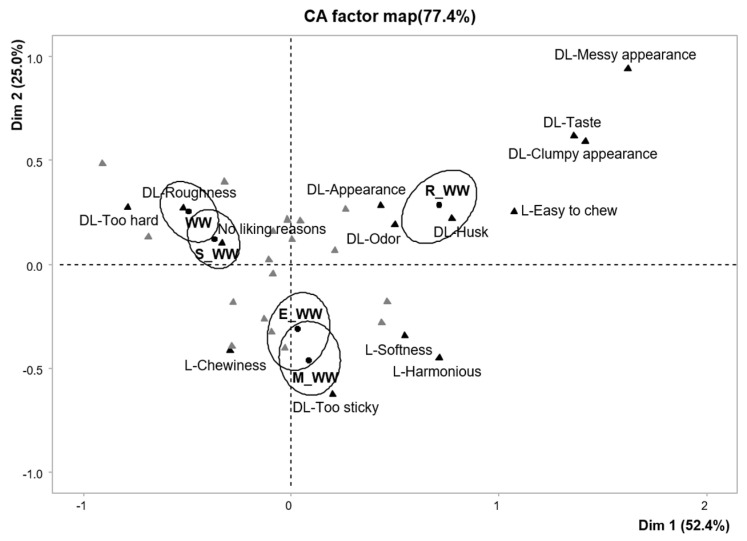
Correspondence analysis plot for reasons of (dis) liking attributes and their corresponding various processed whole wheat sample loadings. Filled circle shapes indicate the sample loadings and filled triangle point up shapes refer to the descriptors.

Figure 1 shows the perceptual map result of correspondence analysis (CA). Dimensions (Dim) 1 and 2 explained 77.4% of the total variation (52.4% and 25.0% of the variation, respectively). The results of CA regarding the reasons for liking and disliking samples were largely divided into three parts. Dim 1 differentiated the cooked rice with R_WW (positive axis) with the lowest overall liking score from the cooked rice with WW and S_WW (negative axis). The subjects commonly mentioned ‘taste’ and ‘odor’ as reasons for disliking the cooked rice with R_WW. In addition, the subjects particularly disliked the cooked rice with R_WW due to the appearance, mentioning terms such as ‘messy appearance’, ‘clumpy appearance’, and ‘husk’. On the other hand, the cooked rice with R_WW was mentioned as being ‘easy to chew’ as a reason for liking. The cooked rice with WW and S_WW was described with ‘too hard’ and ‘roughness’ as reasons for disliking. The cooked rice with R_WW (positive axis), WW, and S_WW (negative axis), separated by Dim 1, had similar overall liking scores, but there were differences in the reasons for liking and disliking these samples. Subsequently, the cooked rice with E_WW and M_WW, with relatively high overall liking scores, was on the negative axis of Dim 2. These samples were associated with the term ‘harmonious’ and texture attributes such as ‘chewiness’ and ‘softness’. However, some subjects mentioned ‘too sticky’ as a reason for disliking these samples. To summarize the finding of the CA results, Dim 1 was mainly defined by reasons of liking and disliking (positive axis: appearance-related attributes, negative axis: texture-related attributes), and Dim 2 was described by overall liking score (positive axis: relatively low, negative axis: relatively high).

## 4. Discussion

The moisture content of a variety of grain kernels such as wheat, brown rice and paddies was maximized by steeping for more than 5 h regardless of temperature [26,27,28]. In this study, the water content of cooked rice with S_WW and E_WW was increased compared to that with WW since S_WW and E_WW were steeped in water for 24 h. As Park et al. [29] and Gujral et al. [30] reported an increased water content with the degree of milling for brown rice, in this study, the water content of cooked rice with M_WW and R_WW was increased as the bran of the wheat kernel surface was destroyed. Texture is the expression of the structure and surface properties of foods through human senses, which affect consumer perceptions of product acceptability [31]. Various instrumental methods have been developed to evaluate the textural properties of foods [32]. Texture profile analysis (TPA) using a texture analyzer is the most frequently used method of applying compression to imitate the mastication process [33,34]. Cohesiveness is a mechanical texture property related to the deformation of food before breaking. The WW sample showed lower cohesiveness than the E_WW sample because it has a fibrous and firm outer layer. Grains with an outer fibrous brown layer, such as brown rice, can prevent the structure from collapsing after the first compression, and the grain retains its shape after compression, resulting in low cohesiveness [30]. Hardness is the first characteristic perceived during the mastication process and shows a good correlation between sensory data and instrumental data in terms of textural characteristic [35]. Kim et al. [36] showed that texture is the most influential characteristics for the overall quality of cooked rice in the sensory evaluation. Particularly, in Kim et al.’s [37] study, which analyzed the correlation between overall sensory quality and TPA of cooked rice, hardness was a negative correlation with overall sensory quality. Texture influenced overall product quality as well as consumer acceptance. Choi et al. [38] reported that stickiness was important for their overall liking of cooked rice. Park et al. [29] reported that the hardness and chewiness tend to decrease as the degree of milling increases. When classifying cooked rice samples, the factors of hardness and stickiness are more than the taste [39], and it was reported that the change in hardness by processing is an important factor when preparing cooked rice using whole wheat with low preference. Normally, the hardness of cooked rice is 2000 g to 3000 g, which is much lower compared to the cooked rice sample with WW [29,40,41]. As the hardness of M_WW and E_WW decreased, the overall consumer liking score and texture liking score increased. The responses to open-ended questions for the cooked rice samples with M_WW and E_WW confirmed the above results by showing that the mechanical texture characteristic of hardness has a substantial influence on sensory evaluation when it is seen that there are few people who dislike it because it is too hard [40].

In this study, consumers were asked to describe the reasons for liking and disliking samples, as previously proposed by Symoneaux et al. [10]. As a result of textual analysis, the number of likes and dislikes was correlated with the overall liking score, and it was possible to predict the level of overall liking. The cooked rice with M_WW with the highest liking score had 128 likes and 97 dislikes, while the cooked rice with R_WW with the lowest liking score had 101 likes and 163 dislikes (Table 3). The higher the liking score of the sample was, the greater the number of likes and the fewer dislikes, and vice versa [10,11]. In addition, when there were more reasons for liking than disliking, the overall liking score was more than 5 points based on the 9-point hedonic scale, and this trend was observed to have the same pattern in Symoneaux et al. [10] which evaluated samples using a 7-point hedonic scale. However, even though the samples had similar liking scores, this may have been caused by different reasons. The cooked rice with WW, S_WW, and R_WW had similar liking scores, but the reasons for liking and disliking these samples were different. For cooked rice with WW and S_WW, a common reason for disliking was ‘too hard’, while this description was never mentioned for cooked rice with R_WW. Appearance characteristics such as ‘messy appearance’, ‘clumpy appearance’, and ‘husk’ were frequently described as reasons for disliking cooked rice with R_WW. Often, when consumers answer only one open-ended question, it can be difficult to know whether what they refer to is positive or negative for them. Asking separately for reasons of liking and disliking makes the transcription of terms easier and provides insight to clearly identify the attributes that are positively and negatively correlated with the consumer’s preference without further interpretation [11,42]. Furthermore, we can identify which attributes are more important to consumers’ preferences, and even the same terms can be liked by some consumers and disliked by others. In conclusion, asking consumers separately for reasons of liking and disliking is a better way to understand their opinion [10].

It is also noteworthy that consumers used more diverse terms to describe the reasons for disliking the samples than liking them. The terms were integrated by three native researchers, obtaining 60 and 80 types of reasons for liking and disliking, respectively. In addition, the chi-square per cell analysis results showed that differences between samples were often larger in terms of dislikes than likes. In previous studies, using open-ended questions [14,43], consumers reportedly cited more likes than dislikes, which was the opposite of this study. The samples surveyed in this study had a relatively lower overall liking score than in previously conducted studies. Alternatively, Letarte et al. [44] reported that dislikes result from more specific and intense sensory experiences than likes. This result may also be due to the positive-negative asymmetry effect in which negative information is weighted more than positive information [45].

Consumers often referred to sensory-related terms rather than holistic or emotional terms. Only three (‘easy to chew’, ‘looks healthy’, and ‘harmonious’) of the 16 reasons for liking and two (‘messy appearance’ and ‘feels undercooked’) of the 16 reasons for disliking mentioned by more than 5% of consumers were non-sensory terms. This is because consumers are relatively familiar with the samples. In fact, 73.8% of consumers who participated in this study responded that they ate multigrain cooked rice more than once a week (data not shown).

The main advantage of open-ended questions is that information about attributes that are important to consumers is collected directly in their own language. A limited number of available attributes in conventional profiling can lead to a dumping effect, and if consumers like the product, a halo effect can occur when evaluating attributes [14]. However, the spontaneity given to consumers in free comment methods allows them to freely describe their sensory perception and emphasize the perceived dominant descriptors. In other words, information about consumers’ perceptions of characteristics can be provided. Nevertheless, open-ended questions still exhibit labor-intensive issues associated with the preprocessing phase, in which spelling errors are eliminated and synonyms are grouped.

## 5. Conclusions

This study was performed regarding the physical properties and consumer acceptance for cooked rice with various types of processed whole wheat. The results of TPA showed that all processed whole wheat samples were less hard than cooked rice with WW, indicating a distinct improvement in the texture property. Thus, consumer acceptance test of these processed whole wheat samples was conducted, with M_WW scoring relatively high overall liking for consumers. This result suggested that M_WW was suitable for cooked rice.

On the other side, two open-ended questions, evaluated separately on the reasons of liking and disliking, confirmed that the sensory characteristics associated with texture were identified as important factors in the formation of acceptance in cooked rice containing various types of processed whole wheat. Among the texture properties, ‘chewiness’ was the driver of liking, while ‘hardness’ and ‘roughness’ were the drivers of disliking. In particular, incomplete appearances such as ‘husk’, ‘clumpy appearance’, and ‘messy appearance’ were drivers of disliking. Additionally, the results of this study imply that the level of overall liking for samples could be predicted by including a separate open-ended question for reasons of liking and disliking.

The present study has several limitations in generalizing results. Although the goal was to verify the marketability of relatively new foods, whole wheat products for cooked rice, comparative analysis with multi grain cooked rice that occupies the market was not particularly investigated in the present study. Further investigations on comparative analysis with marketed products may provide useful insights in developing marketing strategies for these food items.

## Figures and Tables

**Table 1:** Water content, TPA and HI of cooked rice samples with various processed whole wheat.

Sample	Water Content (%) ***^1^	TPA						HI
		Hardness (g) ***	Adhesiveness	Springiness	Cohesiveness ***	Chewiness (g) ***	Resilience **	
WW	57.8 ^c2^	4693 ^a^	−79.2	0.50	0.35 ^b^	810 ^a^	0.16 ^ab^	526
S_WW	60.6 ^b^	3830 ^b^	−85.8	0.48	0.31 ^c^	563 ^bc^	0.13 ^c^	563
M_WW	60.6 ^b^	3822 ^b^	−87.1	0.43	0.32 ^bc^	532 ^bc^	0.14 ^bc^	593
E_WW	60.8 ^b^	3349 ^c^	−82.9	0.47	0.38 ^a^	600 ^b^	0.17 ^a^	596
R_WW	62.3 ^a^	2469 ^d^	−80.2	0.52	0.39 ^a^	505 ^c^	0.18 ^a^	602

TPA: Texture profile analysis, HI: starch hydrolysis index, WW: cooked rice with whole wheat, S_WW: cooked rice with steeped whole wheat, M_WW: cooked rice with milled whole wheat, E_WW: cooked rice with enzyme treated whole wheat, R_WW: cooked rice with rolled whole wheat. ^1^ Significance levels are as follows: (***) *p* < 0.001; (**) *p* < 0.01. ^2^ Mean within a column not sharing a superscript letter are significantly different (*p* < 0.05).

**Table 2:** Mean liking scores of various processed whole wheat samples.

Sample	Overall ***^1^	Appearance ***	Odor ***	Texture ***	Taste/Flavor ***
r^2^	1.00	0.59	0.59	0.74	0.76
WW	4.8 ± 1.9 ^cd^^3^	5.0 ± 1.8 ^b^	5.9 ± 1.6 ^ab^	4.6 ± 2.3 ^b^	5.4 ± 1.8 ^b^
S_WW	5.0 ± 1.8 ^bc^	5.0 ± 1.9 ^b^	5.9 ± 1.5 ^ab^	4.5 ± 2.1 ^b^	5.5 ± 1.7 ^ab^
M_WW	5.8 ± 1.5 ^a^	5.6 ± 1.7 ^a^	6.1 ± 1.5 ^a^	5.7 ± 1.8 ^a^	5.9 ± 1.6 ^a^
E_WW	5.3 ± 1.7 ^b^	5.0 ± 1.7 ^b^	5.5 ± 2.0 ^b^	5.5 ± 1.7 ^a^	5.6 ± 2.0 ^ab^
R_WW	4.4 ± 1.8 ^d^	4.0 ± 1.9 ^c^	4.7 ± 1.9 ^c^	4.6 ± 1.9 ^b^	4.6 ± 2.0 ^c^

WW: cooked rice with whole wheat, S_WW: cooked rice with steeped whole wheat, M_WW: cooked rice with milled whole wheat, E_WW: cooked rice with enzyme treated whole wheat, R_WW: cooked rice with rolled whole wheat. ^1^ Significance levels are as follows: (***) *p* < 0.001. ^2^ Correlation coefficients between overall liking and other likings attributes. ^3^ Mean within a column not sharing a superscript letter are significantly different (*p* < 0.05).

**Table 3:** The frequency of the major reasons of liking and disliking for cooked rice samples by more than 5% of consumer in the free comment as a response to open-ended questions.

Major Driver of (Dis) Liking	Samples					
	WW	S_WW	M_WW	E_WW	R_WW	Total
Reasons of liking						
Appearance	6	4	9	3	1	23
Odor	8	8	4	8	9	37
Corn odor	6	6	4	8	2	26
Nutty odor	5	5	5	2	4	21
Taste	5	4	6	8	3	26
Sweetness	2	6	7	6	3	24
Nutty flavor	6	9	5	3	9	32
Chewiness	16	17	20	25 ^(+)^^1^*^2^	2 ^(−)^***	80
Softness	2	0 ^(−)^*	8	5	8	23
Sticky	4	6	5	3	4	22
Not watery	2	5 ^(+)^*	1	1	0	9
Less after-effect	3	4	5	1	6	19
Easy to chew	0	1	4	0	10 ^(+)^***	15
Looks healthy	6	3	3	2	2	16
Harmonious	0	0	5	3	5	13
No liking reasons	28 ^(+)^*	19	14	13	26	100
Reasons of disliking						
Appearance	9	6	4	10	22 ^(+)^***	51
Husk	0	2	1	3	7 ^(+)^***	13
Clumpy appearance	0	0	1	0	6 ^(+)^***	7
Messy appearance	0	0	0	0	6 ^(+)^***	6
Odor	2	2	2	3	7	16
Off-odor	0 ^(−)^***	3	1	10	7	21
Taste	0	0	0	1	5 ^(+)^***	6
Texture	4	2	3	1	6	16
Too watery	8 ^(−)^***	5 ^(−)^**	29 ^(+)^***	10	25	77
Too hard	33 ^(+)^***	22 ^(+)^*	10	3 ^(−)^**	0 ^(−)^***	68
Roughness	17	18 ^(+)^*	1 ^(−)^***	8	10	54
Too sticky	2	1	8 ^(+)^***	4	3	18
Chewiness	3	5	1	1	1	11
Strong after-effect	14	20	8	11	15	68
Feels undercooked	10 ^(+)^***	1	1	1	0	13
No disliking reasons	13	21	24 ^(+)^***	14	10 ^(−)^***	82
Driver of (dis)liking						
Total reasons of liking	103	109	128	122	101	563
Total reasons of disliking	145	122	97	115	163	642

^1^ (+) or (−) indicate that observed value is higher or lower than the expected value. ^2^ Significance levels are as follows: (***) *p* < 0.001; (**) *p* < 0.01; (*) *p* < 0.05, *p*-value obtain from Chi-square per cell.

## Data Availability

The data to support the findings of this study are included within this article.
